# Application of Machine Learning to the Prediction of Cancer-Associated Venous Thromboembolism

**DOI:** 10.21203/rs.3.rs-2870367/v1

**Published:** 2023-05-08

**Authors:** Simon Mantha, Subrata Chatterjee, Rohan Singh, John Cadley, Chester Poon, Avijit Chatterjee, Daniel Kelly, Michelle Sterpi, Gerald Soff, Jeffrey Zwicker, José Soria, Magdalena Ruiz, Andres Muñoz, Maria Arcila

**Affiliations:** Memorial Sloan Kettering Cancer Center; Memorial Sloan Kettering Cancer Center; Memorial Sloan Kettering Cancer Center; Memorial Sloan Kettering Cancer Center; Memorial Sloan Kettering Cancer Center; Memorial Sloan Kettering Cancer Center; MSK; Mount Sinai Hospital; University of Miami Health System/Sylvester Comprehensive Cancer Center; Memorial Sloan Kettering Cancer Center; Biomedical Research Institute Sant Pau (IIB-Sant Pau); Universidad Complutense; Hospital General Universitario Gregorio Marañón; Memorial Sloan Kettering Cancer Center

## Abstract

Venous thromboembolism (VTE) is a common and impactful complication of cancer. Several clinical prediction rules have been devised to estimate the risk of a thrombotic event in this patient population, however they are associated with limitations. We aimed to develop a predictive model of cancer-associated VTE using machine learning as a means to better integrate all available data, improve prediction accuracy and allow applicability regardless of timing for systemic therapy administration. A retrospective cohort was used to fit and validate the models, consisting of adult patients who had next generation sequencing performed on their solid tumor for the years 2014 to 2019. A deep learning survival model limited to demographic, cancer-specific, laboratory and pharmacological predictors was selected based on results from training data for 23,800 individuals and was evaluated on an internal validation set including 5,951 individuals, yielding a time-dependent concordance index of 0.72 (95% CI = 0.70–0.74) for the first 6 months of observation. Adapted models also performed well overall compared to the Khorana Score (KS) in two external cohorts of individuals starting systemic therapy; in an external validation set of 1,250 patients, the C-index was 0.71 (95% CI = 0.65–0.77) for the deep learning model vs 0.66 (95% CI = 0.59–0.72) for the KS and in a smaller external cohort of 358 patients the C-index was 0.59 (95% CI = 0.50–0.69) for the deep learning model vs 0.56 (95% CI = 0.48–0.64) for the KS. The proportions of patients accurately reclassified by the deep learning model were 25% and 26% respectively. In this large cohort of patients with a broad range of solid malignancies and at different phases of systemic therapy, the use of deep learning resulted in improved accuracy for VTE incidence predictions. Additional studies are needed to further assess the validity of this model.

## Introduction

Cancer has long been known to confer an increased risk of venous thromboembolism (VTE).^1^ The pathophysiological mechanisms are complex and remain incompletely elucidated.^2^ Cancer-associated VTE is common, as approximately 20–30% of VTE episodes are associated with a malignancy.^3^ Those events are clinically important, as they are a leading cause of mortality in patients with cancer.^4^ Several randomized trials have demonstrated the effectiveness of pharmacological prophylaxis. However, applicability has been limited by currently available VTE risk stratification tools.^5,6^ The most commonly used approach to estimate the risk of cancer-associated VTE is the Khorana Score (KS), a clinical prediction rule based on cancer type, peripheral blood cell counts and body mass index.^7^

The KS was originally derived from a cohort of patients who had completed at least one cycle of a new chemotherapy regimen. Using this prediction rule, patients are assigned to one of three categories denoting their risk of VTE at 6 months. The KS has been extensively validated in multiple different healthcare systems.^8^ In one large review, for a KS greater or equal to 2 and 3, sensitivity was 55.2% (95% CI = 47.5%-62.6%) and 23.4% (95% CI = 18.4%-29.4%) respectively, while positive predictive value was 8.9% (95%CI = 7.3%-10.8%) and 11.0% (95% CI = 8.8%-13.8%) respectively.^8^ Several other clinical prediction rules have been derived by different groups.^9-16^ They tend to be based on the predictors already included in the KS, in addition to other clinical or tumor-specific characteristics, routine laboratory test results, presence of germline thrombophilia mutations and chemotherapy administered. In most cases, those algorithms have been derived in patients at the time new systemic therapy was started.

Machine learning (ML) is a computational approach where algorithms are derived automatically from data. In the last decade, ML has found multiple real-world applications including in the medical field.^17^ ML methods are well suited to integrate large amounts of information and derive risk estimation models. Given the proper conditions, ML algorithms can automatically identify complex interactions between predictors which would otherwise be very difficult to elucidate by traditional statistical methods under human supervision. ML will tend to outperform clinical prediction rules, because ML models can easily include multiple predictors and interactions, while clinical prediction rules must be simplistic as they are limited by reliance on human computation.^18^

Recent evidence suggests that tumor somatic genetic alterations influence the risk of VTE.^19-41^ Notably, in some cases gene-specific effects appear to be conditional to tumor type. Additionally, available data seem to indicate an interaction of multiple genes, each contributing a small amount of information to risk prediction, rather than a single gene mediating a large part of the risk. Given those elements of interaction and the need to integrate data on multiple covariates, a ML approach could conceivably help optimize VTE risk prediction based on tumor genomic alterations. In this work, we aimed to derive a ML model to estimate the risk of cancer-associated VTE, incorporating cancer-specific genetic information.

## Methods

### Patient Cohorts

Approval was obtained from the Memorial Sloan Kettering (MSK) institutional review board before initiating this project. The use of data from the ONCOTHROMB 12 – 01 study was authorized by the institutional review board of the Hospital General Universitario Gregorio Marañón (Madrid, Spain). Three cancer patient cohorts were derived: the first one (main MSK cohort) served to train and internally validate the main model, while the two additional sets (external MSK cohort and ONCOTHROMB cohort) were used for external validation and evaluation of transfer learning. The main MSK cohort consisted of all adults who had MSK-IMPACT^™^ (Memorial Sloan Kettering Integrated Mutation Profiling of Actionable Cancer Targets) sequencing performed on their solid tumor malignancy between 2014 and 2019. Patients were included regardless of cancer stage, time from cancer diagnosis or ongoing treatment with anticoagulant or antiplatelet agent. Individuals entered the cohort once their MSK-IMPACT^™^ result was reported in the clinical information system and were censored at the time of their last clinical note. They were included in the analysis without any restriction based on timing for chemotherapy administration, as reporting a more generalizable model was considered desirable. They were excluded if they had sustained an episode of cancer-associated thrombosis before the MSK-IMPACT^™^ result was reported. All sequencing included a patient specific peripheral blood normal control to differentiate between cancer somatic and germline genetic alterations. VTE was defined as pulmonary embolism or lower extremity deep vein thrombosis (DVT). Lower extremity DVT included thrombi involving the common iliac vein, external iliac vein, common femoral vein, superficial femoral vein, deep femoral vein, popliteal vein, peroneal vein, anterior tibial vein, posterior tibial vein or a deep calf vein. All such events were included regardless of the presence of symptoms. A VTE episode was considered cancer-associated if it occurred after or within the 365 days preceding a diagnosis of solid neoplasm. Events were detected using a review of anticoagulant prescriptions, keyword searches of radiology studies and the Clinical Event Detection and Recording System (CEDARS) natural language processing (NLP) pipeline for patients who were included in the cohort between 2014 and 2016, as described elsewhere.^40^ Patients who had MSK-IMPACT^™^ performed between 2017 and 2019 were assessed only using CEDARS as applied to clinical notes and radiology reports.^42^ Briefly, clinical notes and radiology reports were parsed with the spaCy NLP pipeline to derive individual word tokens and associated negation. Documents including any non-negated token combination from a predetermined reference list were presented in chronological order via a custom graphical user interface and reviewed manually. Token combinations for this second CEDARS VTE event detection step are listed in Supplementary Table 1. All detected events were reviewed by two adjudicators, always including a hematologist. A random subset of patients was audited manually to estimate sensitivity and specificity of the automatic event detection algorithms (see Supplementary Information).

The external MSK cohort was aggregated separately for a retrospective study of the association of the KS with overall survival.^43^ Patients were included if they had an active malignancy and were newly started on chemotherapy between October 2017 and November 2019, provided they had sufficient information to compute the KS. All solid tumor types were included. Event detection was conducted using International Classification of Diseases (ICD) 10 codes, a search of pharmacy records for full-dose anticoagulant prescriptions and a text search of radiology reports. VTE was defined as pulmonary embolism or lower extremity DVT. Positive findings were reviewed manually to ascertain the date of a VTE episode. The ONCOTHROMB cohort was prospectively accrued at several hospitals in Spain and was used to derive the TiC-Onco risk-assessment model as part of the ONCOTHROMB 12 – 01 study.^14^ The definition of VTE included pulmonary embolism, lower extremity DVT, superficial vein thrombosis, upper extremity DVT and visceral vein thrombosis. Only individuals with cancer of the esophagus, colon, pancreas or lung were included.

### Model Training, Hyperparameter Tuning and Evaluation of Model Performance

The main MSK cohort dataset was randomly partitioned into a training set comprising 80% of individuals and a validation set with the remainder, stratifying by outcome (VTE or death). The subset of patients who could have their KS calculated was also evaluated separately (“KS subset” of the validation set). In this group, the KS was assessed when an individual was prescribed systemic cancer treatment if this occurred in the first 6 months after MSK-IMPACT^™^ report and there had been no treatment in the past year. The 6-month window restriction was applied to allow for a reliable comparison with models featuring genetic predictors. In the KS subset, predictions were made using laboratory and pharmacy data updated at the time the KS was derived; genomic data was the same as reported in the index IMPACT report.

We used three machine learning algorithms to model the cumulative incidence function of cancer-associated VTE adjusting for the competing risk of death in the main MSK cohort training set: Fine-Gray regression, random survival forests and DeepHit.^44,45^ Details on the choice of algorithm, computing environment, statistical packages and main functions can be found in Supplementary Information. Multivariate feature imputation was used to handle missing data. Continuous predictors used for the DeepHit model were standardized to zero mean and unit variance. Model features were selected based on prior knowledge of their potential contribution to predicting VTE events. Available features could be broadly classified into four groups: basic, lab, chemo and genetic (see Table 1). Those included age, gender, cancer type, metastatic status, time from tumor sampling (i.e. biopsy or surgical resection of tissue sample), time from cancer diagnosis, time elapsed since last systemic therapy administered (stratified by pharmacological class), routine laboratory test results (most recent value available in the prior 3 months for hemoglobin, total protein, albumin, sodium, potassium, chloride, blood urea nitrogen, creatinine, carbon dioxide, glucose, calcium, aspartate transaminase (AST), alanine transaminase (ALT), total bilirubin and alkaline phosphatase), tumor mutational burden and cancer somatic alterations in oncogenes or tumor suppressor genes included in the first generation of the MSK-IMPACT^™^ panel. This assay was described in detail elsewhere.^46^ Only oncogenic or potentially oncogenic alterations were retained, including mutations, copy number alterations and fusions. We decided not to use the white blood count, platelet count, activated partial thromboplastin time and prothrombin time because those values tend to change daily secondarily to influence from chemotherapy (for blood cell counts) and anticoagulation (for clotting times). We felt that even though those predictors might seemingly improve accuracy, the final model could be less generalizable to other healthcare systems with different approaches to laboratory testing. Features were combined into elementary subsets, and the latter were used to derive 11 final feature sets destined to be included in models (see Supplementary Information).

Optimal hyperparameters for random survival forests and DeepHit were determined using a grid search and tree-structured Parzen estimators respectively. Metrics for all three model types were derived using four iterations of five-fold cross-validation, producing 20 values of the metric that were averaged to generate the overall metric. The confidence interval was estimated with bootstrapping. The main metric selected to evaluate models was the time-dependent concordance index as originally derived by Antolini *et al.*^47^ This measurement quantifies the ability of the predictive model to discriminate among subjects with different event times along the cumulative incidence function continuum. An index of 1 indicates perfect concordance between model predicted risk and actual survival, while a value of 0.5 means random concordance. Calibration was assessed with plots of predicted vs observed risk of VTE at 6 months. Observed risk was computed with the Aalen-Johansen estimator in order to account for censoring and the competing risk of death. Patients were categorized in 5 predicted risk group using quantile cutoff points. Models were compared to the KS using the C-index and the concordance/reclassification table. We employed SHapley Additive explanations (SHAP) values to interpret the results of our final machine learning model and gain insights into feature importance and individual predictions. SHAP is a model-agnostic, unified measure of feature importance that builds upon the concept of Shapley values from cooperative game theory.^48^ This approach enables the allocation of a fair contribution of each feature taking into account all possible feature combinations and their marginal contributions to the prediction. In order to ensure the quality of performance report for the risk prediction models described herein, we used the Transparent Reporting of a multivariable prediction model for Individual Prognosis or Diagnosis (TRIPOD) tool (check-list in of Supplementary Table 2).^49^

### Model Validation

The best model was selected based on the C-index and potential usefulness in clinical practice. This model was re-fitted on the whole training set and evaluated on the validation set. All other models were considered secondary. Secondary models designed to account for unavailable predictors were validated on the external MSK cohort and the ONCOTHROMB cohort. We compared those models to the KS in external cohorts using concordance/classification tables. Using the KS, the high-risk group was defined as having a score of 2 or more because this threshold was used in prior studies of pharmacological VTE prophylaxis.^5,6,50^ Risk was dichotomized for the DL models using a threshold of 9% risk of VTE at 6 months, because this was the observed risk for individuals with a KS of 2 or more in a large review.^8^ As a means to further delineate the role of transfer learning in updating VTE prediction models, we evaluated the first secondary model in its original state and after fine-tuning the weights of the output layer on a dedicated transfer learning set from the external MSK cohort.

## Results

### Model Development and Selection

**Figure 1:**
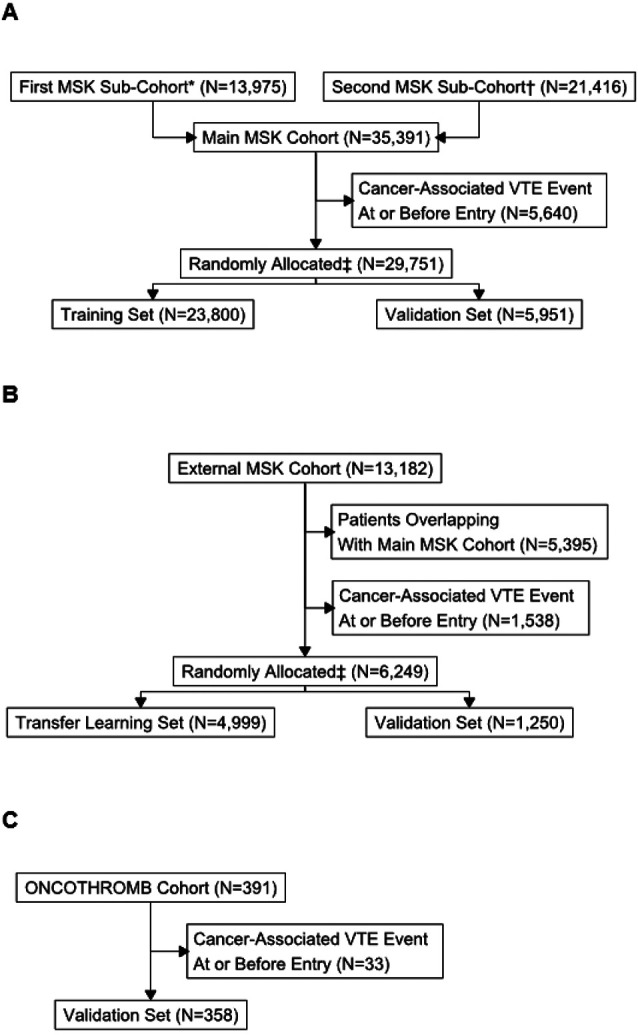
Flow Diagram of Patient Selection for the Three Cohorts A: Main MSK Cohort B: External MSK Cohort C: ONCOTHROMB Cohort *First sub-cohort consisted of adults with blood control drawn for MSK-IMPACT^™^ between 2014 and 2016 †Second sub-cohort consisted of adults with blood control drawn for MSK-IMPACT^™^ between 2017 and 2019 ‡Patients randomly allocated, stratifying by event type.

**Figure 2:**
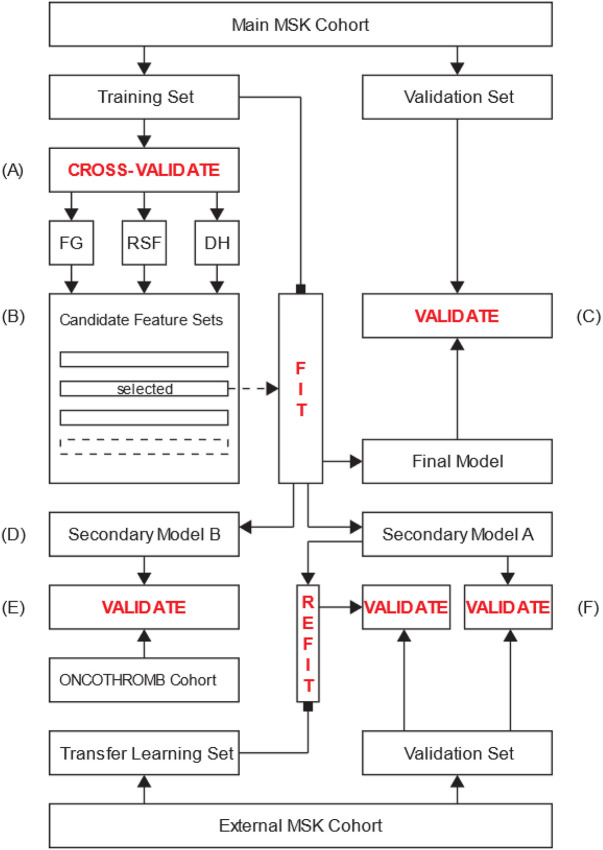
Diagram of Data Flow A: The main MSK cohort training set is utilized to derive and assess the performance of models corresponding to predefined feature sets using five-fold cross-validation. Three machine learning algorithms are evaluated: Fine-Gray competing risk regression (FG), random survival forests (RSF) and DeepHit (DH). B: The models are compared based on their respective C-index and perceived clinical usefulness. The feature set corresponding to the best model is selected and used to derive a new model from the entirety of the main MSK cohort training set. C: This final model is validated on the main MSK cohort validation set. D: Secondary models A and B are derived using the same feature set as derived in (B), excluding features for which the values are unknown in the external MSK cohort and the ONCOTHROMB cohort respectively. E: Secondary model B is validated on the entirety of the ONCOTHROMB cohort. F: Secondary model A is validated on the external MSK cohort validation set. As an exploratory analysis, this model is updated on the external MSK cohort transfer learning set and validated on the corresponding validation set.

**Figure 3:**
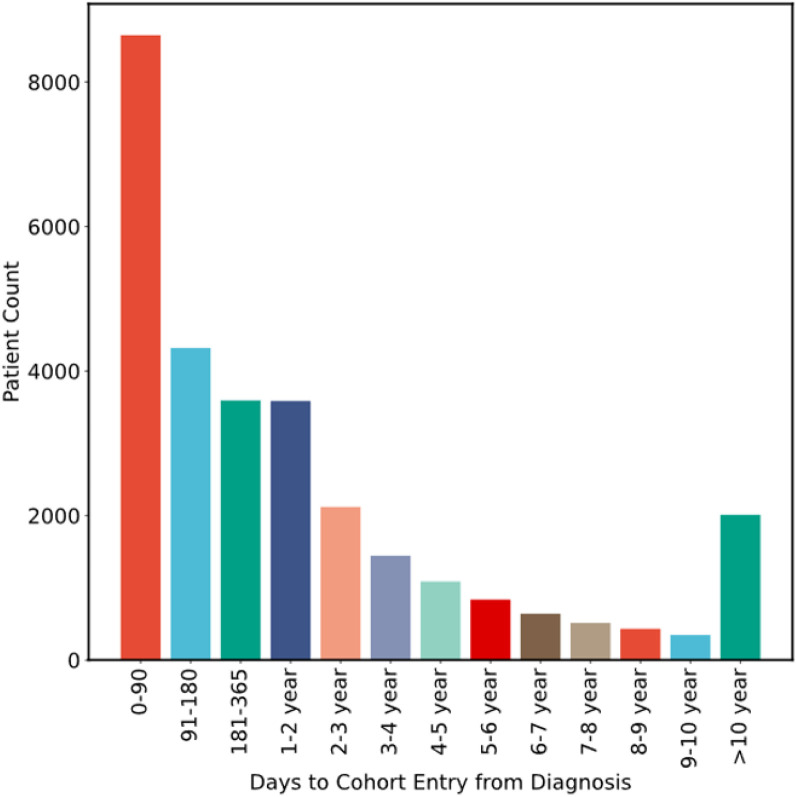
Distribution of Times from Cancer Diagnosis to Main MSK Cohort Entry Cancer diagnosis time corresponds to first pathological evidence of neoplasia and cohort entry is defined by report of MSK-IMPACT^™^ results.

**Figure 4:**
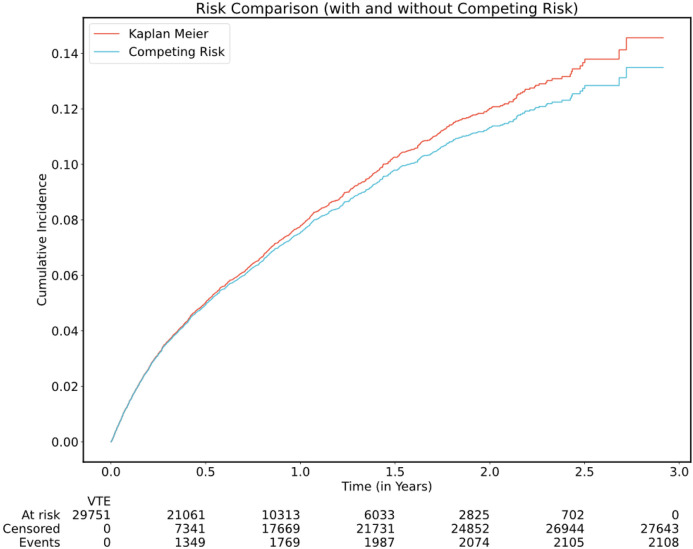
Cancer-Associated VTE Cumulative Incidence Functions in the Main MSK Cohort Cumulative incidence functions were derived from the Kaplan-Meier and the competing risk estimators, the latter using the Aalen-Johansen method.

See Fig. 1 for flow diagram of the selection process for all three cohorts and Fig. 2 for an overview of data flow. A total of 29,751 individuals from the main MSK cohort were included in the final analysis. The characteristics of patients in the main MSK cohort are shown in Table 2. The median age was 62 years. The most frequent tumor type was lung, representing 16% of patients. Less than half of samples were from a metastatic site, with 38% of cases falling into this category. The median time from cancer diagnosis upon cohort entry was 256 days (IQR = 79-1075 days), see Fig. 3. The median observation time was 239 days. Cancer-associated VTE occurred during the first 6 months of observation in 1,338 (4.5%) of the patients. Cumulative incidence functions for this outcome were derived using Kaplan-Meier and competing risk estimators (Fig. 4). The 6-month cumulative VTE estimates using the Kaplan-Meier method and the competing risk estimator were almost identical (5.0% vs. 4.9%), but the difference was more apparent when considering the full observation period (14.6% vs. 13.5%).

Eleven models using distinct covariate sets were derived for each ML approach (see Supplementary Information for detail of the feature sets used). The three approaches (Fine-Gray regression, random survival forests and DeepHit) were applied to each feature set on the main MSK cohort training set (n = 23,800) using five-fold cross-validation. The time-dependent C-index results are provided in Supplementary Table 3. The highest value was noted for the DeepHit model using the “extensive” feature set, including demographics, cancer-specific characteristics, laboratory values, systemic treatment types and genomic predictors (C-index = 0.74, 95% CI = 0.71–0.76). This result was similar to the ones obtained with random survival forests (C-index = 0.73, 95% CI = 0.70–0.76) or Fine-Gray regression (C-index = 0.71, 95% CI = 0.69–0.74). The DeepHit model using the same predictors but excluding genomic information performed similarly (C-index = 0.73, 95% CI = 0.70–0.75). This “limited” set included: age, sex, cancer type, presence or absence of metastatic disease, time from tumor sampling, time from cancer diagnosis, time from last systemic therapy administered for 13 drug classes, albumin, hemoglobin, sodium, potassium, chloride, calcium, carbon dioxide, glucose, urea, creatinine, total protein, AST, ALT, total bilirubin and alkaline phosphatase. Given the absence of a significant improvement in concordance using genomic predictors, we selected the limited feature set. The DeepHit approach was retained, considering that increased complexity was justified by the potential to use transfer learning in the future.

### Internal Validation

**Figure 5:**
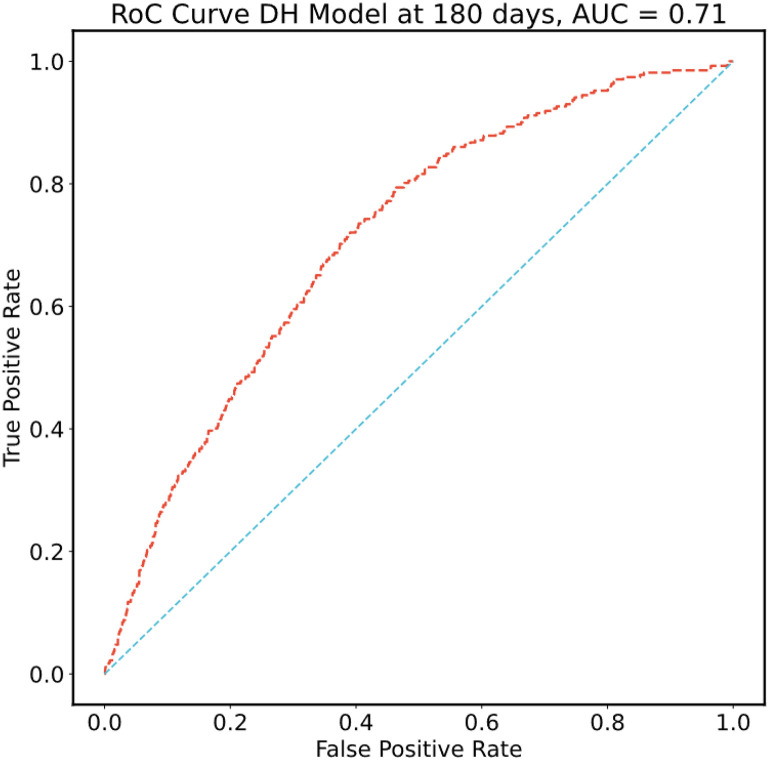
Receiver Operating Characteristic (ROC) Curve for the Selected Model ROC plot computed using the selected DeepHit model featuring a limited set of covariates fitted on the main MSK cohort training set and evaluated in the corresponding validation set.

**Figure 6:**
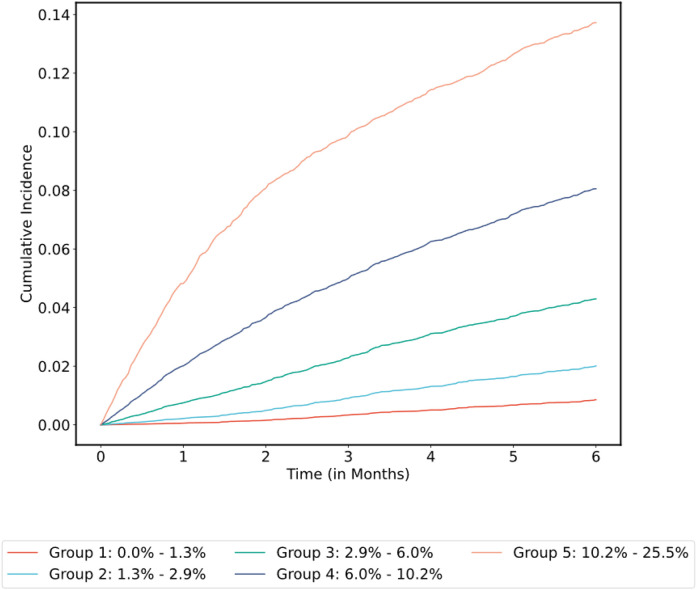
Cumulative Incidence of VTE Stratified by Predicted Risk Group for the Selected Model in the Main MSK Cohort Cumulative incidence functions were derived from the competing risk estimators. Patients grouped by 180-day VTE risk interval based on model prediction and using quantile cutoff points.

Using the optimal hyperparameters derived from cross-validation, all models were re-fitted on the entirety of the main MSK cohort training set and final metrics computed on the corresponding validation set (n = 5,951). Confidence intervals were estimated with bootstrapping. Results using DeepHit for the 11 feature sets are shown in Supplementary Table 4. Using DeepHit and the limited covariate set, the time-dependent C-index was 0.72 (95% CI = 0.70–0.74) on the main MSK cohort validation set. See Fig. 5 for the receiver operating characteristic (ROC) curve and Fig. 6 for the cumulative incidence of VTE stratified by predicted risk group. The calibration plot is shown in Supplementary Fig. 1; predicted risk estimates were outside the confidence interval of the observed risk for only one group. An additional analysis was conducted on individuals who started observation less than one year after their initial cancer diagnosis. In this group of 3,321 patients from the main MSK cohort validation set, the C-index was 0.74 (95% CI = 0.71–0.77) for the DeepHit model featuring a limited set of predictors.

Concordance for the selected model (limited set of covariates) was preserved in the group of patients newly started on systemic therapy (“KS subset” of the main MSK cohort validation set, n = 486), with a time-dependent C-index of 0.74 for the selected DeepHit model (95% CI = 0.67–0.81). The time-dependent C-index using the KS was 0.60 (95% CI = 0.51-.67) for the KS subset of the main MSK cohort validation set. Most patients in the KS subset of the main MSK cohort validation set were at low risk of VTE, as 74% of patients had a KS of 0 or 1 and only 26% had a KS ≥ 2. This is compared to values of 53% and 47% respectively for a large meta-analysis of studies evaluating the KS.^8^

### Inspection of Model Features

**Figure 7:**
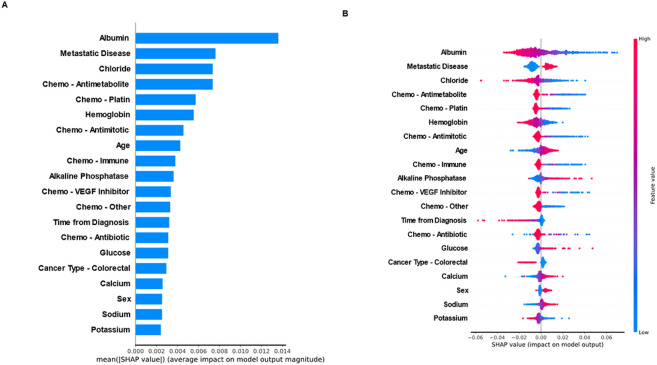
Inspection of Model Features A: Absolute contribution of the 20 features with the highest SHAP values B: Distribution of SHAP values for the 20 features with the highest contribution

The mean absolute SHAP values were calculated for all the features in the selected model (limited set of covariates). See Fig. 7 for the mean values (A) and distribution (B) of the top 20 features. Plasma albumin was the most important feature in the selected DeepHit model, followed by presence of metastatic disease. Several other laboratory values were important predictors of the risk of VTE, including plasma electrolytes (sodium, potassium, chloride and calcium), hemoglobin, glucose and alkaline phosphatase. Higher sodium values, lower chloride and potassium values increased the predicted risk of VTE. Systemic therapy overall was also an important predictor, with a significant effect noted for antimetabolites, antimitotics, antitumor antibiotics, platin analogues, immune checkpoint inhibitors and VEGF inhibitors. Age and sex were among the top 20 features in the model, however only one cancer type was included. The presence of colorectal cancer was associated with a lower risk of VTE compared with other cancer types.

### External Validation and Transfer Learning

**Figure 8:**
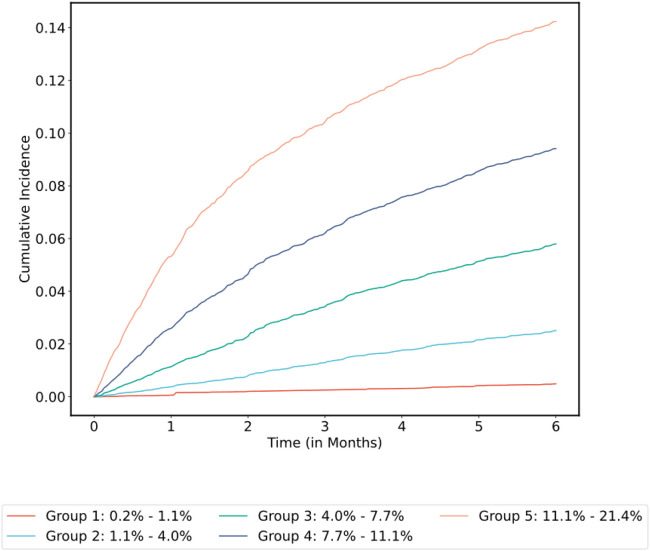
Cumulative Incidence of VTE Stratified by Predicted Risk Group for Secondary Model A in the External MSK Cohort Validation Set Cumulative incidence functions were derived from the competing risk estimators. Patients grouped by 180-day VTE risk interval based on model prediction and using quantile cutoff points.

**Figure 9:**
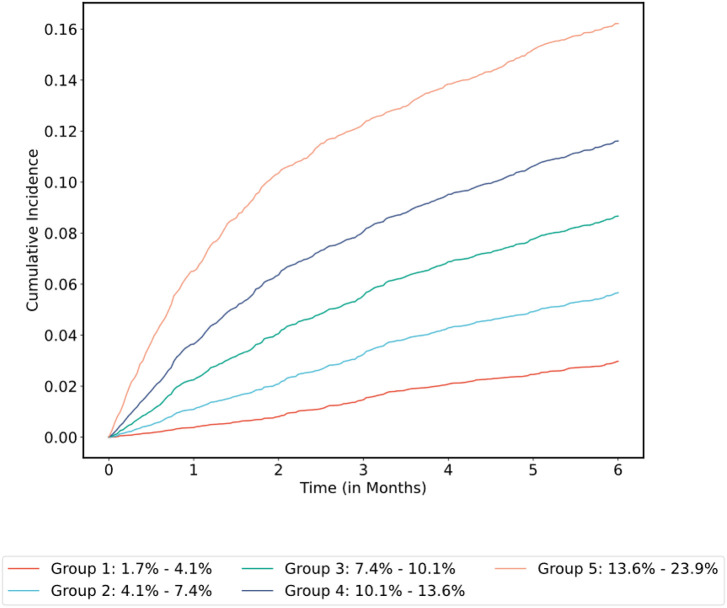
Cumulative Incidence of VTE Stratified by Predicted Risk Group for Secondary Model B in the ONCOTHROMB Cohort Cumulative incidence functions were derived from the competing risk estimators. Patients grouped by 180-day VTE risk interval based on model prediction and using quantile cutoff points.

The external MSK cohort included 6,249 patients and was randomly split in at 4:1 ratio into transfer learning and validation sets. Secondary model A was derived from the main MSK cohort training set using all the features already included in the final model, except for the ones for which values were unknown in the external MSK cohort (metastatic status and time from procedure; see Supplementary Table 5). This model was updated by retraining only the weights of the output layer on the transfer learning set. As a means to assess the added value of transfer learning, the model was also retrained *de novo* on the transfer learning set. The time-dependent C-index was 0.71 (95% CI = 0.65–0.77) for secondary model A before transfer learning, compared with 0.73 (95% CI = 0.66–0.78) after transfer learning and 0.72 (95% CI = 0.65–0.77) for the newly trained model. C-index for the KS was 0.66 (95% CI = 0.59–0.72) on the external MSK cohort validation set. See concordance/classification matrix results in Table 3; 25% of patients were reclassified accurately by secondary model A. See Fig. 8 for the cumulative incidence of VTE stratified by predicted risk group before transfer learning. Calibration plots are shown in Supplementary Fig. 2; observed VTE risk was aligned with model predictions except for one interval for secondary model A before transfer learning (A). All intervals were aligned with observed risk after transfer learning (B). One predicted risk interval group was misaligned with observed risk in the model trained *de novo* (C). The ONCOTHROMB cohort included 358 patients. Secondary model B was derived from the main MSK cohort training set using all the features already included in the final model, except for the ones for which values were unknown in the ONCOTHROMB cohort (systemic therapy type, sodium, potassium, chloride, calcium, carbon dioxide, glucose, urea, total protein, AST, ALT and time from procedure; see Supplementary Table 5). In the ONCOTHROMB cohort, the time-dependent C-index was 0.59 (95% CI = 0.50–0.69) for secondary model B compared to 0.56 for the KS (95% CI = 0.48–0.64). See concordance/classification matrix results in Table 4; 26% of patients were reclassified accurately by secondary model B. See Fig. 9 for the cumulative incidence of VTE stratified by predicted risk group. The calibration plot is shown in Supplementary Fig. 3. Predicted risk estimates were outside the confidence interval of the observed risk for only one group, however the intervals were broad given the limited number of patients in this cohort. Albumin was missing for 30% of individuals in this cohort. In the subset of patients with a reported albumin value (N = 250), The C-index was 0.65 (95% CI = 0.56–0.74) for secondary model B compared to 0.61 (95% CI = 0.52–0.71) for the KS.

## Discussion

This is the first report of a deep learning model for the prediction of cancer-associated VTE. Our approach is novel in several aspects. We have included a broad range of solid tumors and considered a diverse group of patients at all phases of their cancer journey, regardless of systemic treatment status. The latter allows VTE risk estimation for a much larger population of patients than what is currently possible using the KS and related prediction rules, as the majority of risk assessment models reported in the literature only consider patients started on a new chemotherapy regimen, limiting generalizability and applicability to everyday clinical practice. Additionally, we purposefully selected a model estimating the cumulative incidence function of VTE adjusting for the competing risk of death, as opposed to computing event probability at a fixed 6-month time point. This approach minimizes the potential for bias and allows end users to compute the risk of VTE at any arbitrary time point during the validated observation period, providing added flexibility in clinical applications.

The network architecture of the final model is conducive to the application of transfer learning, a methodology which has been studied and applied extensively for neural networks.^51^ We evaluated transfer learning with one external dataset. In this case, there was only a trend toward improvement in model accuracy, arguably because concordance and calibration were already excellent with the original model; this limited the ability of transfer learning to improve prediction modeling. Transfer learning could potentially improve calibration when the model is ported to different cohorts and attenuate inconsistencies in absolute risk estimates, a problem identified in several studies evaluating the KS.^52^ This approach opens the door to a new paradigm for the prediction of cancer-associated VTE, a world where models can be adapted to new healthcare settings in order to maximize external validity.

ML has been used by several groups to predict cancer-associated VTE, with encouraging results.^53-57^ The models presented in those reports were limited to a combination of demographic, cancer-specific and routine laboratory assay predictors. This is the first attempt to use ML to estimate the risk of CAT based on somatic genomic predictors in a large cohort of individuals with a solid tumor. There was no significant benefit to adding genomic predictors to a model already including demographic, cancer-specific, laboratory and pharmacological predictors in the cohort including all individuals regardless of systemic therapy status. These findings suggest that even though cancer somatic genetic alterations contain information about the risk of VTE, redundance exists with other predictors and there is a point at which adding more covariates yields no marginal benefit. The gene-specific information was limited to a binary marker for oncogenes and tumor suppressor genes. It is possible that future work using more granular information (e.g. alteration type, variant allele frequency) and including other genes (e.g. coagulation factors, cytokines) would result in improved prediction accuracy. Interestingly, albumin was the most important feature in the final model. An association between a decreased serum albumin level and an increased risk of cancer-associated VTE has been reported previously.^58^ On the other hand, while plasma electrolyte levels were important features in the model, those markers have not been previously reported to be associated with the risk of cancer-associated thrombosis.

Concordance was preserved in the KS subset of the main MSK cohort validation set for the selected deep learning model using a set of covariates including widely available clinical, pathological and laboratory predictors. Concordance for this model was superior to what was obtained using the KS. Such satisfactory performance in the subset of patients starting systemic therapy was confirmed with a similar model in the larger external MSK cohort validation set. The latter findings are important because this group of patients commencing cancer treatment is currently the focus of pharmacological VTE prophylaxis and has been featured prominently in other studies of a predictive model.

The main limitation of this work is the retrospective nature of the model derivation cohort. Relying on medical records can affect sensitivity and increases the risk of bias in capturing events of interests. However, for a cohort of this size (29,751 individuals) prospective VTE event capture would be prohibitively costly. We feel satisfactory precautions have been taken to ensure reliability of event capture for this cohort. Notably, the cumulative incidence of VTE was consistent with values reported in other studies, suggesting a low rate of missed cases. VTE cases were identified in the main MSK cohort using a novel NLP workflow, which can conceivably be more sensitive than the use of billing data to find relevant clinical events. Data missingness for covariates is unavoidable for large cohorts and can be problematic when attempting to fit predictive models. The possible consequences in this case include decreased model accuracy and more rarely a biased model if missingness is informative and not accounted for properly during imputation. In this regard, we used multivariate feature imputation which uses the entire set of available features to estimate the missing values. Also, missing data was not common and limited to laboratory predictors (missingness between 10%-14% for most of the values with only the carbon dioxide predictor missing for 36% of its values), so the impact on the final model is expected to be low in the MSK cohorts. However, the substantial rate of missingness noted for albumin in the ONCOTHROMB cohort might have contributed to inferior performance of the DeepHit model for which this laboratory value was an important feature. Ultimately the value of the final model will greatly depend on its external validity, i.e. its performance in other healthcare systems. As discussed in a recent set of guidelines for the standardization of risk prediction model reporting in cancer-associated thrombosis, additional work will be necessary before implementation in other healthcare systems.^59^

## Conclusion

VTE is an important complication of cancer for which effective pharmacological prophylaxis methods exist. Currently available prediction rules have limited accuracy in stratifying patients for VTE risk. Future avenues to improve the overall benefit of VTE prophylaxis in this group will be contingent on better methods to quantify risk, a task for which ML is well-suited. The work presented here suggests that deep learning for survival analysis can be used to estimate the risk of cancer-associated VTE with accuracy. Future external validation studies are needed to assess generalizability of the model derived with this cohort. The use of genomic predictors and transfer learning should be further explored and developed.

## Figures and Tables

**Table 1:** Overview of Features Used in the Models

Group	Feature	Description
basic	age	age in years at time of cohort entry
	sex	male or female
	cancer type	bladder, breast, colorectal, esophagogastric, gynecological, head and neck, hepatobiliary, high-grade glioma, low-grade glioma, lung, melanoma, pancreatic adenocarcinoma, prostate adenocarcinoma, renal, soft tissue sarcoma or other
	metastatic disease	presence or absence of metastasis
	time from tumor sampling	time in days since biopsy or resection of MSK-IMPACT^™^ sample
	time from cancer diagnosis	time in days since cancer was first diagnosed
lab	most recent laboratory value in the prior 3 months (15 individual predictors)	albumin, hemoglobin, sodium, potassium, chloride, calcium, carbon dioxide, glucose, urea, creatinine, total protein, AST, ALT, total bilirubin, alkaline phosphatase
chemo	time from last systemic treatment (13 individual predictors)	time in days since last systemic anti-cancer treatment for each of alkylating, antibiotic, antimetabolite, antimitotic, cyclin-dependent kinase inhibitor, epidermal growth factor receptor inhibitor, immune, multikinase inhibitor, PARP inhibitor, platin, SERM, VEGF inhibitor or other; capped at 28 days, equal to zero if drug prescribed but not yet administered
genetic	presence of a cancer somatic genetic alteration (55 individual predictors)	binary marker indicating the presence or absence of an oncogenic or potentially oncogenic alteration (mutation, copy number alteration or fusion) in each of 55 genes found to have an alteration frequency ≥ 1.5% (see Supplemental Information for gene list)
	tumor mutational burden	number of somatic missense mutations per megabase (Mb) of tumor genome

AST: aspartate transaminase

ALT: alanine transaminase

PARP: poly ADP-ribose polymerase

SERM: selective estrogen receptor modulator

VEGF: vascular endothelial growth factor

**Table 2:** Characteristics of Patients

	Main MSK Cohort			External MSK Cohort	ONCOTHROMB Cohort
	Overall	Training Set	Validation Set		
Characteristic	N = 29,751	N = 23,800	N = 5,951	N = 6,249	N = 358
Age in Years, Median (IQR)	62 (52, 71)	62 (52, 71)	62 (52, 70)	64 (53, 72)	65 (57, 72)
Female Sex, N (%)	16,424 (55)	13,101 (55)	3,323 (56)	3,637 (58)	118 (33)
Time From Cancer Diagnosis in Days, Median (IQR)	256 (79, 1,075)	255 (80, 1,067)	262 (77, 1,108)	67 (37, 199)	42 (24, 65)
Time From Tumor Sampling in Days, Median (IQR)	75 (39, 257)	75 (39, 258)	74 (39, 254)	NA	NA
WBC in Thousand Cells/mcL, Median (IQR)	6.5 (4.9, 8.6)	6.5 (4.9, 8.6)	6.4 (4.9, 8.5)	7.1 (5.8, 8.8)	8.0 (6.6, 10.0)
Missing, N	2,942	2,365	577	17	0
Hemoglobin in g/dL, Median (IQR)	12.1 (10.7, 13.3)	12.1 (10.7, 13.3)	12.1 (10.8, 13.3)	12.9 (11.6, 13.9)	12.9 (11.4, 14.0)
Missing, N	2,939	2,363	576	17	0
Platelet Count in Thousand Cells/mcL, Median (IQR)	231 (180, 293)	231 (180, 293)	232 (181, 296)	253 (205, 311)	279 (220, 343)
Missing, N	2,945	2,368	577	20	0
Albumin in g/dL, Median (IQR)	4.0 (3.7, 4.3)	4.0 (3.7, 4.3)	4.0 (3.7, 4.3)	4.2 (3.9, 4.4)	4.0 (3.7, 4.3)
Missing, N	3,846	3,094	752	197	108
TMB Score in Mutations/Mb, Median (IQR)	4.40 (2.2, 7.0)	4.4 (2.2, 7.0)	3.9 (2.2, 7.0)	NA	NA
Missing, N	1,374	1,065	309	NA	NA
Cancer Type, N (%)					
Bladder	1,033 (3.5)	845 (3.6)	188 (3.2)	567 (9.1)	0
Breast	4,226 (14.0)	3,346 (14)	880 (15.0)	1,822 (29.0)	0
Colorectal	3,103 (10.0)	2,527 (11.0)	576 (9.7)	470 (7.5)	155 (43.3)
Esophagogastric	947 (3.2)	778 (3.3)	169 (2.8)	246 (3.9)	66 (18.4)
Gynecological	2,926 (9.8)	2,336 (9.8)	590 (9.9)	514 (8.2)	0
Head and Neck	643 (2.2)	513 (2.2)	130 (2.2)	680 (11.0)	0
hepatobiliary	857 (2.9)	704 (3.0)	153 (2.6)	138 (2.2)	0
High-Grade Glioma	913 (3.1)	743 (3.1)	170 (2.9)	4 (< 0.1)	0
Low-Grade Glioma	269 (0.9)	207 (0.9)	62 (1.0)	1 (< 0.1)	0
Lung	4,776 (16.0)	3,739 (16.0)	1,037 (17.0)	518 (8.3)	84 (23.5)
Melanoma	1,125 (3.8)	920 (3.9)	205 (3.4)	139 (2.2)	0
Other	3,974 (13.0)	3,166 (13.0)	808 (14.0)	408 (6.5)	0
Pancreatic Adenocarcinoma	1,342 (4.5)	1,096 (4.6)	246 (4.1)	365 (5.8)	53 (14.8)
Prostate Adenocarcinoma	1,974 (6.6)	1,572 (6.6)	402 (6.8)	164 (2.6)	0
Renal	590 (2.0)	463 (1.9)	127 (2.1)	129 (2.1)	0
Soft Tissue Sarcoma	1,053 (3.5)	845 (3.6)	208 (3.5)	84 (1.3)	0
Metastatic Disease, N (%)	11,225 (38)	8,993 (38)	2,232 (38)	NA	155 (43)

**Table 3:** Comparison Between the Khorana Score and Secondary Model A in the External MSK Cohort

	Khorana Score*	DeepHit Model Risk Group†	No.	Incidence of VTE at 6 months‡ (%)
Concordant	Low-Risk	Low-Risk	743	3
	High-Risk	High-Risk	196	19
Reclassified	Low-Risk	High-Risk	158	11
	High-Risk	Low-Risk	153	5

* High-risk group includes patients with a Khorana Score greater or equal to 2.

† High-risk defined as a predicted cumulative incidence of VTE at 6 months of 9% or greater, using Secondary model A

‡ Observed risk of VTE at 6 months computed using the Aalen-Johansen estimator

**Table 4:** Comparison Between the Khorana Score and Secondary Model B in the ONCOTHROMB Cohort

	Khorana Score*	DeepHit Model Risk Group†	No.	Incidence of VTE at 6 months‡ (%)
Concordant	Low-Risk	Low-Risk	137	8
	High-Risk	High-Risk	127	16
Reclassified	Low-Risk	High-Risk	48	13
	High-Risk	Low-Risk	46	7

** High-risk group includes patients with a Khorana Score greater or equal to 2.

† High-risk defined as a predicted cumulative incidence of VTE at 6 months of 9% or greater, using Secondary model B

‡ Observed risk of VTE at 6 months computed using the Aalen-Johansen estimator

## Data Availability

The MSK data that support the findings of this study are available from the corresponding author upon reasonable request. The dataset for the ONCOTHROMB cohort is not openly available.

## Key Points

1. Deep learning for survival analysis can be used to accurately estimate the risk of cancer-associated venous thromboembolism.
2. The newly developed time-dynamic model is distinguished from prior models which are commonly restricted to baseline testing prior to systemic therapy.

## Code Availability

The code is available online at https://github.com/MOTREC-AI/ML-CAT.
